# Intravitreal administration of recombinant human opticin protects against hyperoxia-induced pre-retinal neovascularization

**DOI:** 10.1016/j.exer.2021.108908

**Published:** 2022-02

**Authors:** Izabela P. Klaska, Anne White, Pilar Villacampa, Justin Hoke, Laura Abelleira-Hervas, Ryea N. Maswood, Robin R. Ali, Catey Bunce, Richard D. Unwin, Garth J.S. Cooper, Paul N. Bishop, James W. Bainbridge

**Affiliations:** a UCL Institute of Ophthalmology, University College London, 11-43 Bath Street, London, EC1V 9EL, UK; b KCL Centre for Cell and Gene Therapy, Tower Wing, Guy's Hospital, Great Maze Pond, London, SE1 9RT, UK; c Division of Evolution & Genomic Sciences, School of Biological Sciences, FBMH, University of Manchester, Manchester, UK; d Josep Carreras Leukaemia Research Institute, Ctra de Can Ruti, Barcelona, Spain; e School of Population Health and Environmental Sciences, Faculty of Life Sciences and Medicine, King's College London, Addison House, London, SE1 1UL, UK; f Division of Cardiovascular Sciences, School of Medical Sciences, FBMH, University of Manchester, Manchester, UK; g Stoller Biomarker Discovery Centre and Division of Cancer Sciences, School of Medical Sciences, FBMH, University of Manchester, Manchester, UK; h Manchester Royal Eye Hospital, Manchester University NHS Foundation Trust, Manchester Academic Health Science Centre, Manchester, UK

**Keywords:** Opticin, Angiogenesis, Neovascularization, OIR, PDR, ROP

## Abstract

Opticin is an extracellular glycoprotein present in the vitreous. Its antiangiogenic properties offer the potential for therapeutic intervention in conditions such as proliferative diabetic retinopathy and retinopathy of prematurity. Here, we investigated the hypothesis that intravitreal administration of recombinant human opticin can safely protect against the development of pathological angiogenesis and promote its regression. We generated and purified recombinant human opticin and investigated its impact on the development and regression of pathological retinal neovascularization following intravitreal administration in murine oxygen-induced retinopathy. We also investigated its effect on normal retinal vascular development and function, following intravitreal injection in neonatal mice, by histological examination and electroretinography. In oxygen-induced retinopathy, intravitreal administration of human recombinant opticin protected against the development of retinal neovascularization to similar extent as aflibercept, which targets VEGF. Opticin also accelerated regression of established retinal neovascularization, though the effect at 18 h was less than that of aflibercept. Intravitreal administration of human recombinant opticin in neonatal mice caused no detectable perturbation of subsequent retinal vascular development or function. In summary we found that intraocular administration of recombinant human opticin protects against the development of pathological angiogenesis in mice and promotes its regression.

## Highlights

• Human recombinant opticin protects against the development of pathological angiogenesis in mice.
• Human recombinant opticin accelerates regression of established pre-retinal neovascularization in mice.
• Intraocular administration of recombinant human opticin offers the potential for a safe and effective therapy for neovascular retinal diseases.

## Abbreviations

AV: avascular area
EC: endothelial cell
ECM: extracellular matrix
ERG: electroretinography
N: number
NV: pre-retinal neovascularization
P: postnatal day
PBS: phosphate buffered saline
PDR: proliferative diabetic retinopathy
ROP: retinopathy of prematurity
RT: room temperature
SEM: standard error of the mean
OIR: oxygen-induced retinopathy
VEGF: vascular endothelial growth factor
VO: vaso-obliteration

## 1 Introduction

Retinopathy of prematurity (ROP) and proliferative diabetic retinopathy (PDR) are both characterized by retinal ischemia and pathological retinal neovascularization (Wheatley et al., 2002; Antonetti et al., 2012). ROP is a major cause of sight impairment in children (Wheatley et al., 2002; Hartnett and Penn, 2012; Tin and Gupta, 2007) and PDR is a leading cause in adults (Antonetti et al., 2012; Das et al., 2015). Conventional management by pan-retinal photocoagulation (PRP) is of limited efficacy and causes predictable ocular adverse effects including impairment of peripheral retinal function (Hartnett and Penn, 2012; Das et al., 2015). Although intravitreal administration of vascular endothelial growth factor (VEGF) inhibitors, such as aflibercept, can provide valuable benefit, this can be limited in extent and duration. In neonates, anti-VEGF agents may harm development of the eye and other organs (Tokunaga et al., 2014). In adults with diabetes, prolonged use of anti-VEGF agents is associated with a possible increased risk of death and cerebrovascular events (Avery and Gordon, 2016).

The vitreous is a gel that occupies the posterior segment of the eye where its optical transparency is critical for normal vision. The mature vitreous is normally avascular and virtually acellular. It contains a network of collagen fibrils that maintain the gel state but, in certain conditions including retinopathy of prematurity and proliferative diabetic retinopathy, serves as a scaffold for the development of pathological neovascularization (Bishop, 2015). Vitreous contains anti-angiogenic factors, including pigment epithelial-derived factor (PEDF), thrombospondin-1, and endostatin, that help maintain its avascular state. Opticin is a 90 kDa homodimeric extracellular matrix glycoprotein, a class III member of the small leucine-rich repeat protein (SLRP) family, that was first identified in association with vitreous collagen fibrils (Reardon et al., 2000; Le Goff et al., 2003). Opticin is secreted into the vitreous cavity by non-pigmented ciliary epithelium. Immunolocalization studies have demonstrated its association with vitreous collagen fibrils and internal limiting membrane (Takanosu et al., 2001; Bishop et al., 2002; Ramesh et al., 2004), though in the rabbit vitreous greater than 90% of opticin is present in the vitreous supernatant and not bound to collagen fibrils pelleted after centrifugation of the vitreous gel (Del Amo et al., 2020). Opticin acts as an endogenous inhibitor of angiogenesis that inhibits angiogenesis stimulated by a range of different growth factors (Le Goff et al., 2012; Le Goff et al., 2012). Angiogenesis requires the presence of extracellular ligands such as collagen, fibrin or fibronectin to support the process, and endothlelial cells use specific integrins to interact with these ligands (Bishop, 2015). On binding to collagen, opticin disrupts its interactions with α1β1 and α2β1 endothelial cell integrins and its effects on angiogenesis are matrix dependent. It inhibits angiogenesis by interfering with integrin-mediated endothelial cell collagen interactions, but does not interfere with intergrin-mediated endothelial cell interactions with fibronectin or vitronectin (Le Goff et al., 2012a, Le Goff et al., 2012b).

In mice with oxygen-induced retinopathy, which is a model of the ischemia-induced retinal neovascularization that characterises retinopathy of prematurity and proliferative diabetic retinopathy, endogenous opticin protects against pathological retinal neovascularization (Le Goff et al., 2012a, Le Goff et al., 2012b). Furthermore, supplementation of endogenous opticin with recombinant bovine opticin administered into the vitreous provides additional protection (Le Goff et al., 2012a, Le Goff et al., 2012b). In the present study, we investigated the hypotheses firstly that intravitreal administration of recombinant human opticin can protect against the development of retinal neovascularization, and secondly that it can promote regression of established retinal neovascularization in murine oxygen-induced retinopathy.

## 2 Materials and methods

### 2.1 Generation of recombinant opticin

Recombinant human opticin was expressed in 293-EBNA cells, purified from conditioned media, formulated in PBS buffer, and tested for endotoxin contamination as described previously (Del Amo et al., 2020).

### 2.2 Animal experiments

We used the contralateral eye of each animal as a control that was un-injected. We measured the area of neovascularization and the area of avascular retina by quantification of immunofluorescence in retinal flat-mounts stained by fluorescein-conjugated isolectin B4. All animal studies were carried out under the Animals (Scientific Procedures) Act 1986 (project license PPL 70/8120, protocol number 5). All procedures were conducted following ethical approval of the Animal Welfare and Ethics Committee of the UCL Institute of Ophthalmology and complied with the ARVO Statement for the Use of Animals in Ophthalmology and Vision Research. C57BL/6J mice were obtained from the Harlan (UK) and maintained in the animal facility at the UCL Institute of Ophthalmology (standard 12/12 h light-dark cycle). All efforts were made to minimize the number of animals used and their distress.

### 2.3 Mouse model of oxygen-induced retinopathy (OIR)

To induce OIR, mice litters were exposed to 85% oxygen from postnatal day 8 (P8) until P11, when they were returned to normoxic atmospheric conditions (room air). The number of mice used for each experiment was calculated according to previous data (Villacampa et al., 2017). Owing to the limited capacity of the oxygen chamber, we combined data from independent experiments. The oxygen in the hyperoxia chamber was regulated by a ProOx110 controller (BioSpherix; Parish, NY, USA). Pups from each litter were randomly allocated to each experimental group and in the same fashion the treatment was assigned to left or right eye. Given a recognized association between low postnatal weight gain and the course/severity of OIR (Stahl et al., 2010), only pups with body weight of 5g or greater were included. Under general anaesthesia, pups were injected intravitreally (1 μl total volume) using an 8 mm 34-gauge needle (Hamilton; Nevada, USA) advanced through the sclera into the vitreous under direct vision using an operating microscope (Carl Zeiss; Germany). In each animal we injected one eye only and used the un-injected contralateral eye to control for the variability in responses between animals to OIR; we expressed the outcome for each individual animal as the ratio of areas in its injected and un-injected eyes. To control for the recognized nonspecific effect of injection/needle trauma in OIR, we compared the mean value of the opticin group with the mean value of the group injected with PBS. As a positive control, we also evaluated the effect of aflibercept for its established antiangiogenic properties. To investigate the effect on the development of retinal neovascularization, we performed injections at P11, the day of onset of retinal ischemia, and measured the outcome at P16, the peak of induced neovascularization. To investigate the effect on regression of established neovascularization, we performed injections at P16 and assessed the outcome 18 h later before spontaneous regression of neovascularization is advanced. In all experiments, mice were euthanized by cervical dislocation. Enucleated globes were fixed immediately by immersion in 4% (w/v) paraformaldehyde (PFA) for 1 h.

### 2.4 Immunofluorescence

Following fixation, the retinas were dissected and incubated in blocking buffer (PBS containing 5% normal goat serum, 1% bovine serum albumin and 1% Triton X-100) for 1 h at room temperature (RT). Next, to visualise the vasculature, the retinas were incubated overnight at 4 °C in biotin-conjugated Bandeiraea simplicifolia isolectin B4 antibody (iB4, 1:200; Sigma-Aldrich), followed by Alexa Fluor 546-conjugated streptavidin secondary antibody (1:500; Invitrogen by Thermo Fisher) incubation for 2 h at RT. Following staining, the retinas were flat-mounted on microscope slides using Fluorescent Mounting Medium (Dako; Carpinteria, CA, USA).

### 2.5 Imaging and quantification

Retinal flatmounts were imaged by confocal microscopy (Leica TCS SPE, Leica Microsystems). Mosaic stack images were acquired to visualise the entire retinal vasculature at 512 × 512 pixel resolution with a 10 × dry objective. Stacks were Z-projected using the Leica LAS AF confocal software and images were quantified in a masked fashion using ImageJ software (NIH, US). Having selected and measured total retinal area, the avascular retinal region (AV) corresponding to vaso-obliterated (VO) retinal area was demarcated and quantified using the “Freehand” selection tool. The extent of pre-retinal NV was quantified by manual selection of individual neovascular tufts and clusters using the threshold function.

### 2.6 Safety studies

To investigate whether opticin perturbs retinal vascular development or function we injected it intravitreally into neonatal mouse pups at P2 (hypothermia was used to induce anaesthesia; their eyelids were opened by incising the developing palpebral aperture; one eye was injected and the contralateral eye was used as an un-injected control). Animals were culled at P10 and dissected retinas were stained with isolectin B4 antibody to visualise vasculature (see section 2.4 for details). To calculate vascular density, we measured the number of pixels of lectin fluorescence in 3 areas per retina and expressed this as a proportion of the total retinal area. Branching points were quantified in 3 areas per retina, the values represent number of branching points per 10000 square μm. Confocal scans were set up to construct z stacks including all planes containing positive staining. At P28 (26 days post injection) pups were examined by electroretinography (see below) prior to culling.

### 2.7 Electroretinography (ERG)

ERG responses were recorded using Espion ERG Diagnosys System (Diagnosys) and acquired data were analysed using Espion software. Scotopic recordings were obtained from animals dark-adapted overnight at the following increasing light intensities: 0.000001, 0.00001, 0.0001, 0.001, 0.01, 0.1, 1, 10, 31.6 and 75.28 cd s/m^2^. Photopic recordings were performed following 5 min light adaptation intervals on a background light intensity of 30 cd s/m^2^, which was also used as the background light for the duration of photopic recordings. Photopic light intensities used were: 0.01, 0.1, 1, 10, 31.6 and 75.28 cd s/m^2^.

### 2.8 Statistical analysis

We powered the study statistically on the basis of our own prior experience of oxygen-induced retinopathy. To detect a 20% change in neovascularization with power 80% and probability 95% in a 2-sided test, we calculated that a minimum of 8 animals would be required for each experimental group. Treatment allocation was randomised, and assessment of outcomes was fully masked. Data were analysed using GraphPad Prism (version 5.04) and Stata (version 14.1) software. For data distributed normally, differences between groups were measured by un-paired t-tests. To explore differences between specific experimental groups we performed no adjustment for multiple comparisons. Data sets were examined for evidence of non-normality using Shapiro-Wilk test. Skewed data were log-transformed prior to subsequent analysis. The numbers of animals and the statistical test used for individual experiments are described in the Figure legends.

## 3 Results

### 3.1 Opticin protects against the development of retinal neovascularization and reduces the avascular area in accelerated OIR

**Fig. 1:**
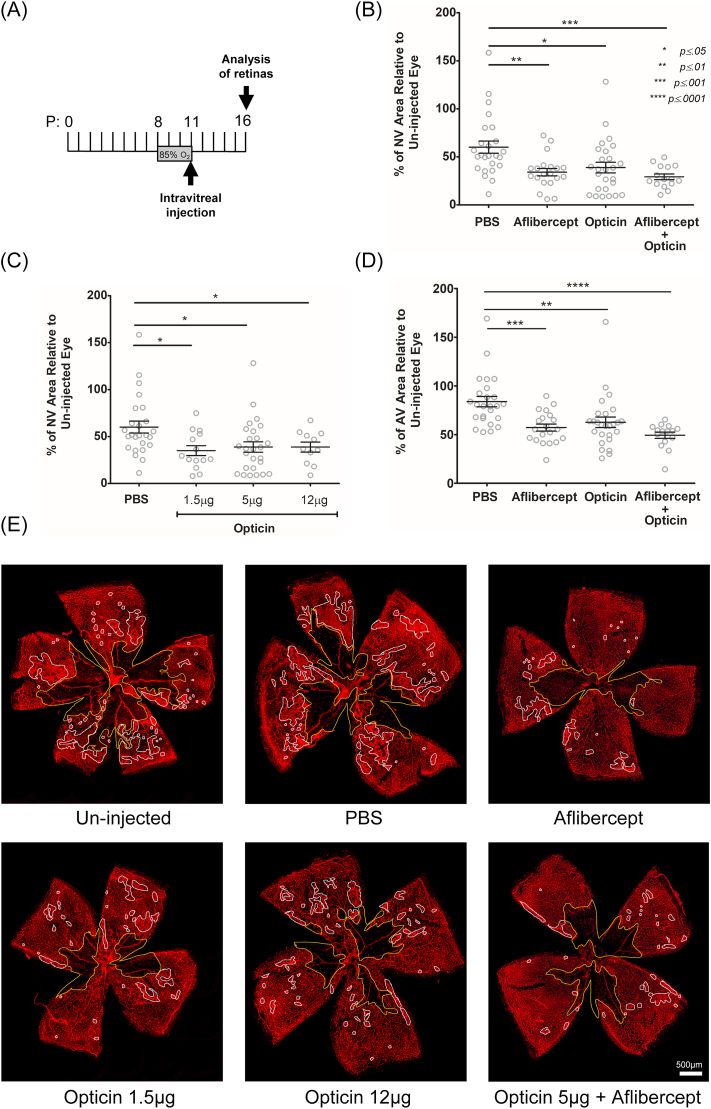
**Opticin protects against the development of retinal neovascularization and reduces the avascular area in accelerated OIR.** (A) C57BL/6 pups were subjected to 85% oxygen from P8 to P11, followed by normal atmospheric conditions. Opticin, aflibercept (2 μg) or PBS were injected intravitreally at P11 (one eye injected; fellow eye used as internal un-injected control), pups were culled at P16 and dissected retinas were stained with isolectin B4 to visualise the retinal vasculature. (B) Effects of opticin (5 μg) on pre-retinal neovascularization using accelerated OIR model. (C) Opticin dose response analyzing effect on neovascularization. (D) Effects of opticin (5 μg) and aflibercept (2 μg) on area of avascular retina. (E) Representative images of flat-mounted retinas at P16; the avascular area delineated in yellow, and the area of pre-retinal neovascularization delineated in white. Scale bar 500 μm. Number of mice within each experimental group were as follows: PBS = 25, aflibercept = 20, opticin 1.5 μg = 14, opticin 5 μg = 26, opticin 12 μg = 11, aflibercept + opticin 5 μg = 15. The percentage (%) of neovascular area (NV)/avascular area (AV) area was measured against total area of flat-mounted retina. Histograms show the extent of NV and AV retina in the injected eye relative to the un-injected contralateral eye (mean ± SEM). Differences between the groups were evaluated using two-tailed un-paired t tests (*p ≤ .05, **p ≤ .01, ***p ≤ .001, ****p ≤ .0001). . (For interpretation of the references to colour in this figure legend, the reader is referred to the Web version of this article.)

Murine oxygen-induced retinopathy (OIR) is a model of ischemia-induced retinal neovascularization, widely used to investigate underlying pathological mechanisms and to develop therapeutic interventions (Villacampa et al., 2017; Smith et al., 1994). Exposure of pups to hyperoxia during development induces regression of areas of the immature retinal vasculature (Villacampa et al., 2017). On return to room air, the resulting local ischemia and tissue hypoxia induce both appropriate regeneration of the retinal vasculature and aberrant growth of neovascular tufts, which extend pre-retinally from the surface of the inner retina into the vitreous before regressing spontaneously. The pre-retinal neovascularization is similar in nature to the pathological neovascularization responsible for impairment of sight in people affected by retinopathy of prematurity and proliferative diabetic retinopathy. To investigate the therapeutic potential of opticin in protecting against pathological neovascularization we used an accelerated version of OIR in which exposure of mouse pups to 85% oxygen from P8 to P11 induces greater regression of the developing retinal vasculature and reliably (Villacampa et al., 2017) induces retinal neovascularization in a shorter time period than the widely used OIR model using 75% oxygen from P7 to P12 (Le Goff et al., 2012a, Le Goff et al., 2012b; Villacampa et al., 2017) (Fig. 1A).

We found that intravitreal injection of recombinant human opticin at P11 protected against hyperoxia-induced neovascularization compared to controls that had received an injection of PBS (Fig. 1B). Opticin (5 μg) reduced the extent of neovascularization by a mean (SEM) of 61% (±5.51; N = 26), whereas PBS reduced the neovascularization by a mean (SEM) of 40% (±6.382; N = 25). This difference in outcome between opticin and PBS was statistically significant (p = .0148) (Fig. 1B and Fig. S1B). In dose-ranging experiments we found no statistically significant differences in the effects of opticin at doses of 1.5 μg, 5 μg and 12 μg on pre-retinal neovascularization (Fig. 1C). Injection of aflibercept (2 μg) reduced neovascularization by 66% (±3.88; N = 20; p < .01) (Fig. 1B and Fig. S1B). Co-administration of human opticin (5 μg) with aflibercept (2 μg) resulted in no measurable additive effect on neovascularization (29.2 ± 2.9%; N = 15), compared to either administered individually (Fig. 1B).

Injection of human recombinant opticin at P11 in OIR reduced the extent of avascular retina at P16 (62.7 ± 5.4%; N = 26) compared to PBS-injected controls (83.9 ± 5.3%; N = 25), indicating accelerated revascularization. This difference in outcome between opticin and PBS was statistically significant (p < .01). Injection of aflibercept (2 μg) also accelerated revascularization in OIR (57.3 ± 3.6%; N = 20; p < .001) (Fig. 1D and Fig. S1C). Co-administration of human opticin (5 μg) with aflibercept (2 μg) resulted in no measurable additive effect on revascularization (49.4 ± 3.31%; N = 15), compared to either agent administered individually (Fig. 1D and E).

### 3.2 Opticin accelerates regression of established/pre-existing pathological neovascularization

**Fig. 2:**
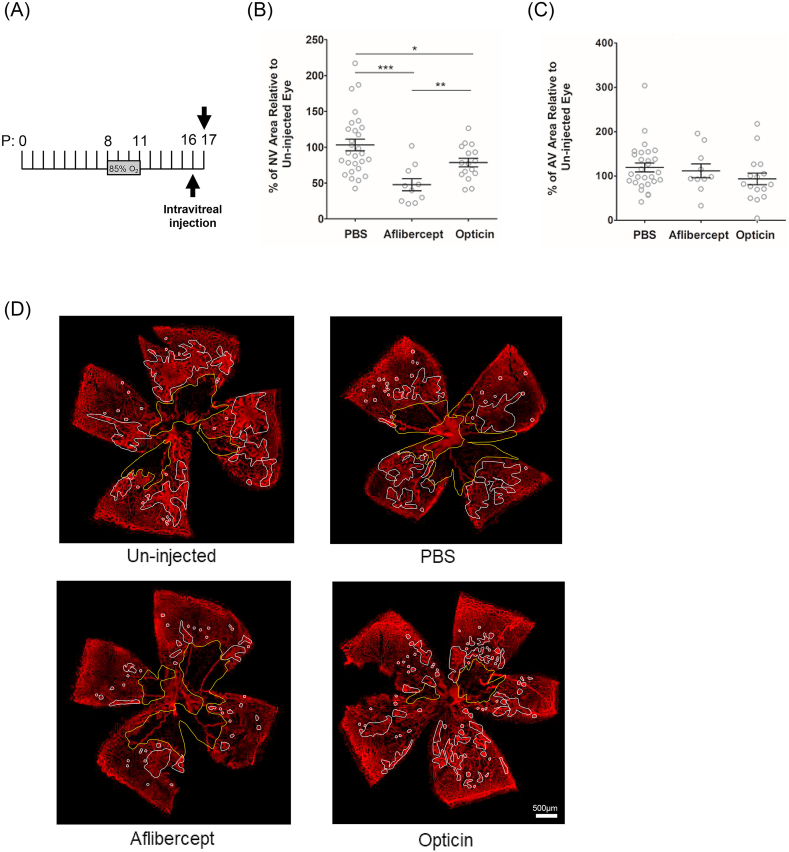
**Both opticin and aflibercept promote regression of retinal neovascularization.** (A) OIR was induced by exposure to 85% oxygen from P8 until P11. At the peak of neovascularization (P16), pups were injected with opticin (12 μg), aflibercept (2 μg) or PBS. 18 h later (on P17), the areas of neovascular and avascular retina were measured. (B) Aflibercept induced 55.5% regression of pre-retinal neovascularization compared to PBS (*p < .0001*). Opticin induced a 24.6% regression compared to PBS (*p* = *.0483*). (C) Neither opticin nor aflibercept altered the avascular area significantly, compared to PBS, 18 h after administration. Histograms show the extent of neovascular (NV) and avascular (AV) retina in the injected eye relative to the un-injected contralateral eye (mean ± SEM). Number of mice within each experimental group were as follows: PBS = 28, aflibercept = 10, opticin 12 μg = 16. Group differences were evaluated using two-tailed un-paired *t*-test (*p ≤ .05, **p ≤ .01, ***p ≤ .001). (D) Representative images of flat-mounted retinas at P17; the avascular area delineated in yellow, and the area of pre-retinal neovascularization delineated in white. Scale bar 500 μm. (For interpretation of the references to colour in this figure legend, the reader is referred to the Web version of this article.)

To investigate the potential of opticin to induce regression of established neovascularization we used a modified version of the standard OIR model (Liu et al., 2013). In OIR pre-retinal neovascularization regresses spontaneously by 50% within 3 days (Villacampa et al., 2017; Connor et al., 2009). We sought to determine whether opticin accelerates this spontaneous regression. We chose to intervene at the peak of neovascularization (P16) and to assess the outcome 18 h later (Fig. 2A). We performed the experiments in parallel and, since the effect of the 1.5 μg dose on neovasculariztion had not been determined at the time, we elected to use the highest concentration available to determine the maximum effect on neovascular regression. One outlying data point was excluded from the analysis owing to inadequate induction of neovascularization in the un-injected control eye (opticin group). Since there was some evidence of skewness in the data (p = .002), the data were log transformed for subsequent analysis. Intravitreal delivery of recombinant human opticin (12 μg) accelerated the regression of pathological pre-retinal neovascularization by a mean (SEM) of 21.39% (±5.987; N = 16) whereas injection of PBS slowed regression of neovascularization by a mean (SEM) of 3.2% (±8.119; N = 28). The difference in outcome between opticin and PBS was statistically significant (p = .05), (Fig. 2B and Fig. S2B). Aflibercept accelerated regression of pathological neovascularization by a mean (SEM) of 52.26% (±8.422; N = 10) (p < .0001). The effect of aflibercept on neovascular regression at P17 was significantly greater than that of opticin (p < .01). We found no measurable effect of opticin or aflibercept on the area of avascular area compared to that of the PBS-injected control group (Fig. 2C and D and Figs. S2C and S2D).

### 3.3 Opticin appears to cause no perturbation of normal retinal vascularisation or function

**Fig. 3:**
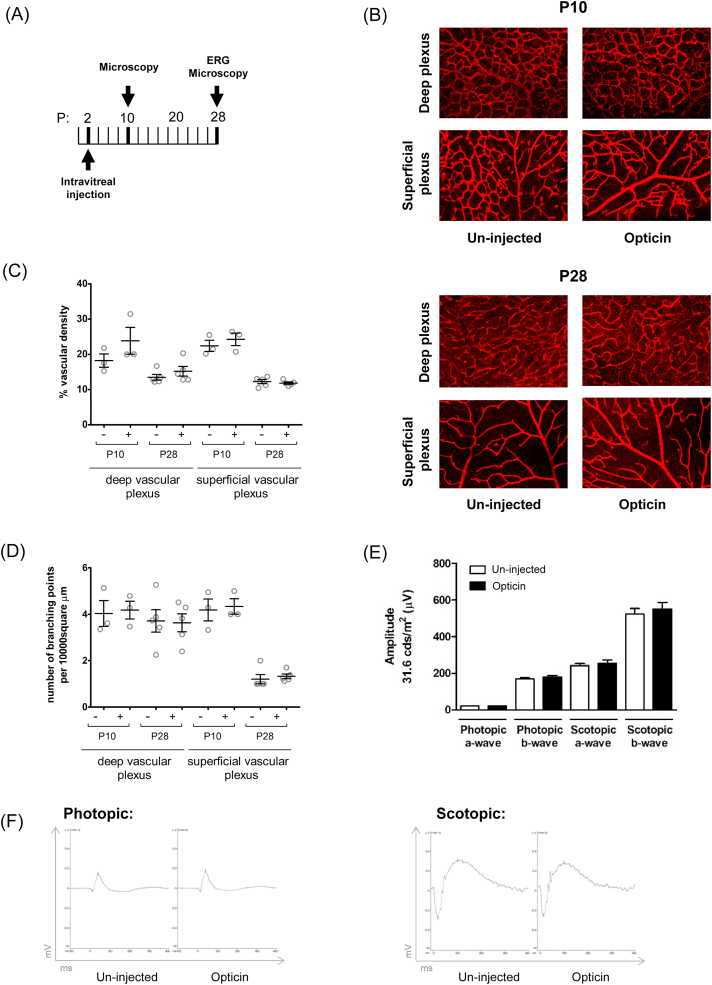
**Opticin does not perturb retinal vascularisation or function.** (A) Human opticin (6 μg in a total volume of 0.5 μl) was administered by intravitreal injection to mouse pups at P2 (1 eye injected, fellow eye used as an un-injected internal control). Mice were culled at P10 and P28 for assessment of retinal vascular development by confocal microscopy of retinal flat mounts. (B) Representative images of deep and superficial vascular plexus at P10 (top panel) and P28 (bottom panel) which did not differ between opticin injected and control eyes. (C) Quantification of vascular density in opticin injected versus un-injected controls. (D) Quantification of vessel branch points in opticin injected versus un-injected controls. (E) Electroretinography was performed 26 days following injection. Multiple white-light flash intensity series were performed, with light intensity increasing in 10 steps (from 0.000001 to 75.28 cd s/m2) for scotopic examination and in 6 steps (from 0.1 to 75.28 cd s/m2) for photopic conditions. Data presented represent ERG responses to stimuli at 31.6 cd s/m^2^. Quantification of scotopic and photopic a- and b- wave responses shows no measurable difference in retinal function. (F) ERG traces from representative mouse (31.6 cd s/m^2^). Data displayed on the graphs represent mean ± SEM. Number of mice within each experimental group were as follows: P10 = 3, P28 = 5. Group differences were evaluated using two-tailed Mann-Whitney test with p < .05 considered as statistically significant.

To investigate the safety of intraocular administration of recombinant human opticin, we examined its effect on the normal development of the major retinal vasculature and retinal function after intravitreal injection of 6 μg dose at P2 (Fig. 3A). We identified no adverse impact on retinal vascular density or number of branch points at P10 (N = 3) and P28 (N = 5) both for deep and superficial vascular plexus (Fig. 3B, C, D). We detected no impact on retinal function as measured by scotopic and photopic a-wave and b-wave amplitudes on electroretinography performed at P28 (Fig. 3 E, F).

## 4 Discussion

Using accelerated OIR, we found that local supplementation of endogenous opticin by intravitreal injection of recombinant human opticin at P11 protects against hyperoxia-induced pathological pre-retinal neovascularization at P16. Intravitreal injection of PBS alone reduces the extent of neovascularization compared to un-injected contralateral eyes. This finding is well-established and is believed to result from an alteration in the balance of pro-angiogenic and anti-angiogenic cytokines such as PEDF owing to local tissue trauma (Tokunaga et al., 2014; Penn et al., 2006). Nevertheless, effects mediated by opticin were significantly greater than those in the PBS control group. We found that, in OIR, opticin appears as effective in protecting against retinal neovascularization as the experimental control aflibercept, a therapeutic anti-VEGF molecule in common clinical use. In dose-ranging experiments we found no clear dose-response of opticin, suggesting complete inhibition of vitreous collagen-binding in OIR even at the lowest dose.

Aflibercept is a recombinant fusion protein that binds VEGF-A, VEGF-B and placental growth factor (PIGF), but not VEGF-C. Both VEGF-A and PlGF promote the growth of abnormal blood vessels during pre-retinal neovascularization. Aflibercept acts as a soluble decoy receptor preventing VEGF from binding and activating its cognate receptors, VEGFR-1 and VEGFR-2. Although VEGFR-2 is the main signal transducer for angiogenesis (signalling via PLCγ-PKC-MAPK pathway) (Koch and Claesson-Welsh, 2012), VEGFR-1 binds VEGF-A with higher affinity, thereby acting as a negative regulator of VEGFR-2 (Shibuya, 2011). VEGFR-2 activity is also modified by the interactions with integrins, transmembrane molecules that mediate cell-matrix adhesion by binding to ECM. VEGF signalling via VEGFR-2 increases integrin dependent migration and reduces the stability of vascular endothelial cadherin adhesions (Carmeliet et al., 1999). Since currently available anti-VEGF agents such as aflibercept can cause harm through dose-related toxicity (Avery and Gordon, 2016) and the adverse effects of repeated intraocular injections, we sought to determine whether local administration of recombinant human opticin offers an alternative with advantages in terms of its anti-angiogenic efficacy, safety and its half-life in the eye. We found that, like aflibercept, recombinant human opticin protects against the development of pathological neovascularization and induces regression of established neovascularization. Although our experiments were not designed specifically to determine whether opticin is as effective as aflibercept, the protective effect against neovascularization appeared similar. However, the effect of opticin on regression of neovascularization at 18 h appeared to be less that than of aflibercept. The mechanistic explanation for this differential effect on development and regression of neovascularization is unknown. We speculate that opticin induces regression less rapidly than aflibercept because it does not perturb endothelial cell interactions with fibronectin.

We found that co-administration of opticin with aflibercept did not result in a greater reduction of neovascularization compared to either agent administered alone. Although opticin and aflibercept act via different target mechanisms, both ultimately interrupt interactions between vascular endothelial cells and the extracellular matrix. By binding VEGF-A, aflibercept interrupts signal transduction through VEGFR-2, abolishing activation of ERK1/2 and p38-MAPK which are important for endothelial cell proliferation and integrin-dependent migration. On binding to collagen, opticin disrupts its interactions with α1β1 and α2β1 endothelial cell integrins. Integrins support VEGF signal transduction, and α1β1 and α2β1 integrins are required for VEGF driven chemotaxis of endothelial cells (Senger et al., 2002). However, opticin does not interfere with integrin-mediated endothelial cell interactions with fibronectin, which are involved in both retinal vascularisation in development and revascularization in OIR (Le Goff et al., 2012a, Le Goff et al., 2012b; Uemura et al., 2006; Maier et al., 2007). This is consistent with our finding that intraocular administration of opticin appears not to affect retinal vascular development adversely.

We found that administration of opticin at the onset of retinal hypoxia reduced the avascular area 5 days later compared to PBS-injected controls, indicating accelerated revascularization of the avascular retina. We found that aflibercept had a similar effect in accelerated OIR, a finding that contrasts with a previous report in standard OIR in which aflibercept (at doses of 2.5 μg and 10 μg) appeared to slow revascularization (Tokunaga et al., 2014). The reason for this difference in findings is unclear but may reflect differences between the standard and accelerated models of OIR, and the time-points of intervention and outcome assessment.

## 5 Conclusions

Although several pro-angiogenic molecular mediators have been identified as possible targets for therapeutic intervention, to date only anti-VEGF molecules have been shown to promote regression of established neovascularization (Liu et al., 2013). Here we report that recombinant human opticin protects against the development of neovascularization and promotes regression of established neovascularization but spares normal retinal vascular development. We have determined that recombinant human opticin has a half-life in the vitreous of 4 days (Del Amo et al., 2020), which compares favourably with that of aflibercept. The results of our investigation in OIR demonstrate no specific advantage of recombinant human opticin over aflibercept. In this model its protective effect against pre-retinal neovascularization is similar, and its effect on regression of established new vessels appears less rapid. However, since its mechanism of action is different, opticin might offer the potential for benefit in specific clinical scenarios, for example where the response to aflibercept is unfavorable. Our findings support the hypothesis that intraocular administration of recombinant human opticin offers the potential for a safe and effective therapy for neovascular diseases of the retina including retinopathy of prematurity.

## Funding

The present study was supported by the 10.13039/501100000265Medical Research Council (grant no. MR/M025365/1).

## Declarations of interest

None.
