# Supplementary figures and images for: Intravitreal administration of recombinant human opticin protects against hyperoxia-induced pre-retinal neovascularization
Source: Exp Eye Res. 2022 Feb;215:108908. doi: 10.1016/j.exer.2021.108908 (PMC8935380; doi:10.1016/j.exer.2021.108908)

## Slide 1
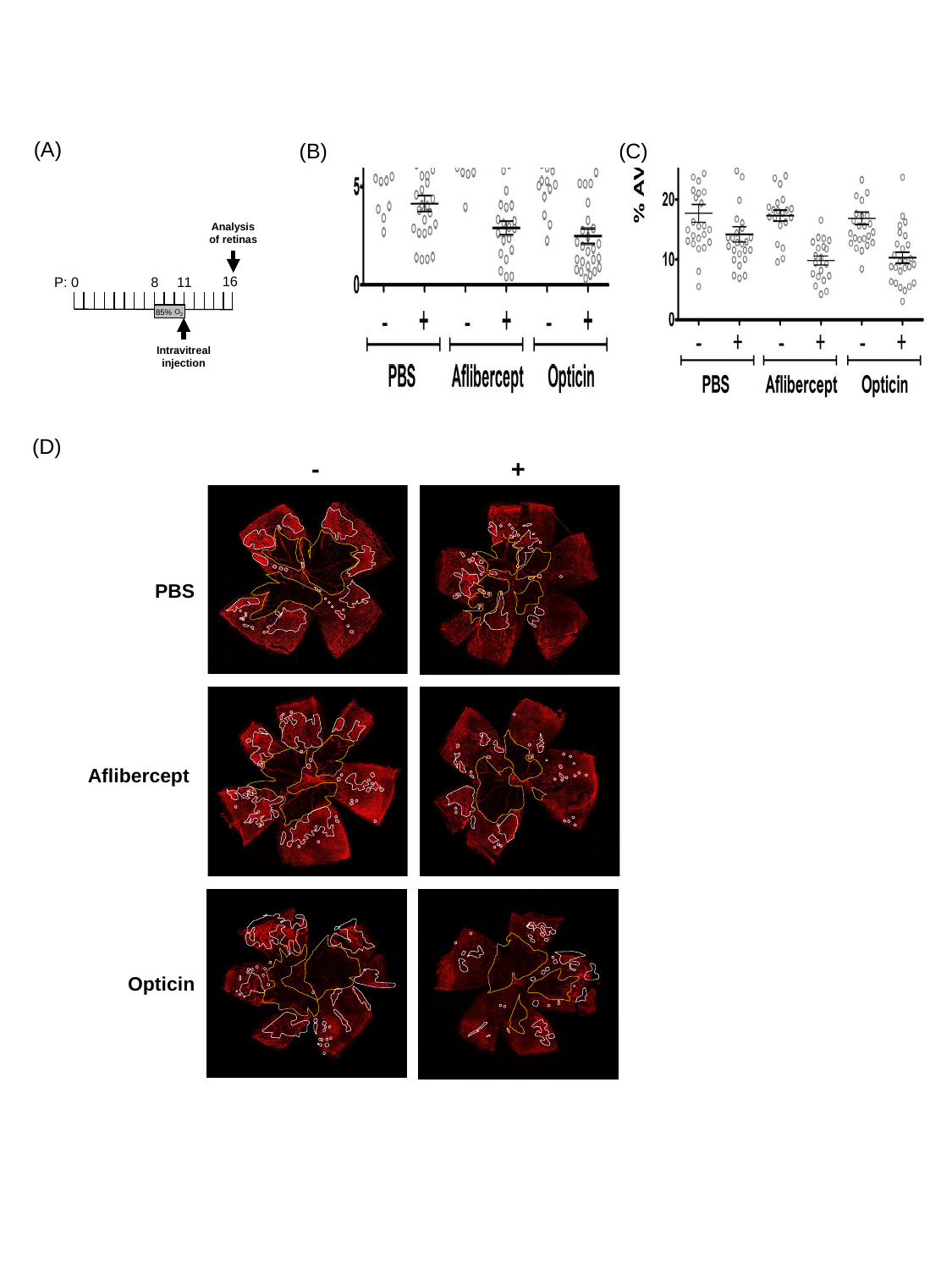

(A)
(B)
(C)
(D)
-
+
PBS
Aflibercept
Opticin

Supplement: Multimedia component 1 [file mmc1.pptx]

## Slide 1
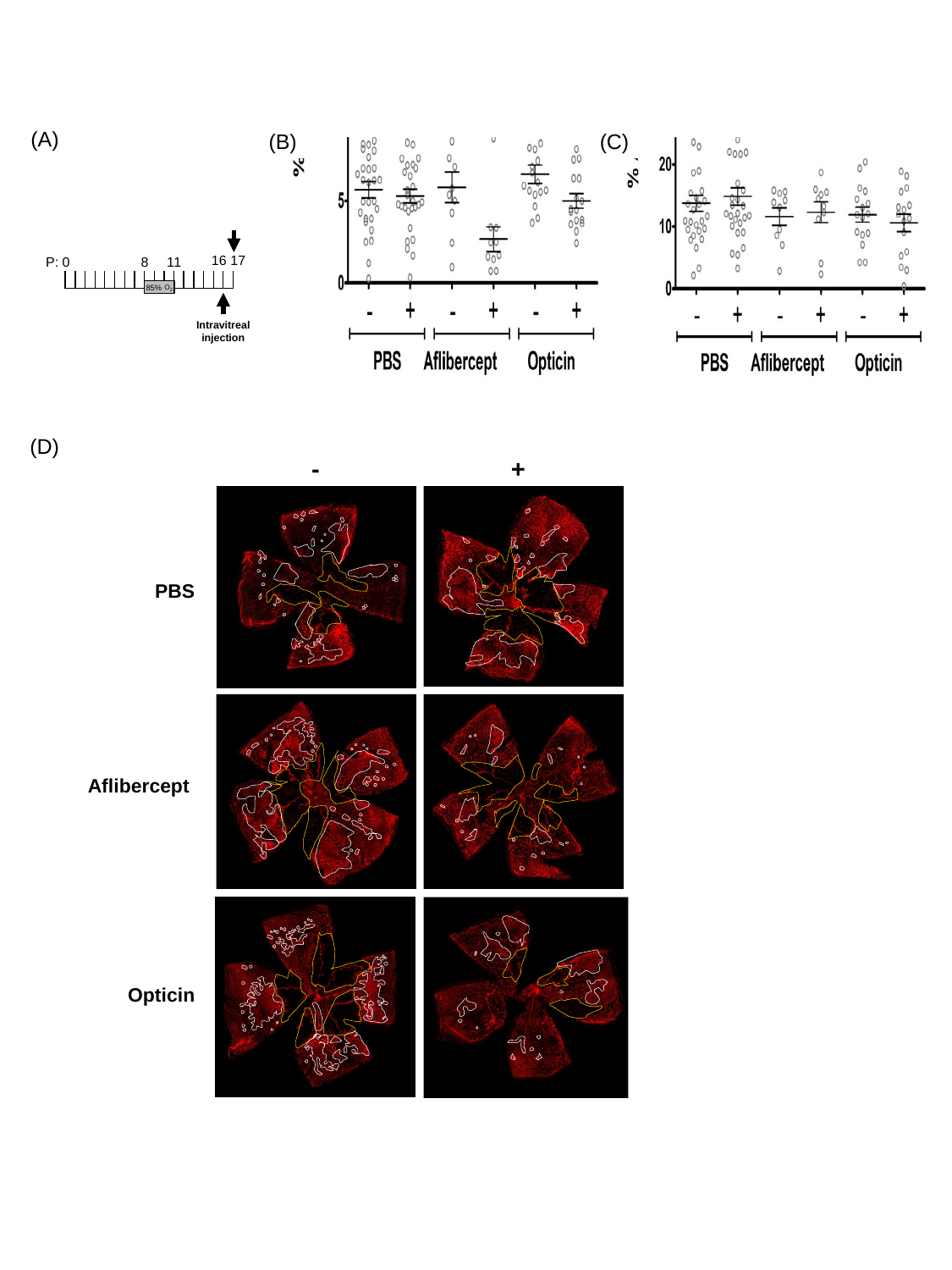

(A)
(B)
(C)
(D)
(D)
-
+
PBS
Aflibercept
Opticin

Supplement: Multimedia component 2 [file mmc2.pptx]
